# A framework to evaluate public green spaces with an emphasis on recreational values for sustainable urban and rural areas: A systematic review and content analysis

**DOI:** 10.1016/j.heliyon.2024.e41553

**Published:** 2025-01-06

**Authors:** Amirmohammad Ghavimi, Frank Schuessler, Roland Pesch

**Affiliations:** Jade University of Applied Sciences, Institute for Applied Photogrammetry and Geoinformatics, Ofener Str. 16, Oldenburg, 26129, Lower Saxony, Germany

**Keywords:** Public green space, Green space, Green space evaluation, Sustainability, Recreational value, Green space qualities

## Abstract

Though numerous studies acknowledge the critical role played by green spaces (GS) in bolstering sustainability in various dimensions, a majority of these investigations primarily center on the ecological aspect and urban environments. Due to the multifaceted benefits of GSs, different categories and expectations of these spaces can be identified across disciplines. Hence, no single method exists for evaluating the success of GSs in promoting sustainability due to the multifaceted benefits and variety of expectations. This study provides a framework for evaluating public green spaces (PGS) holistically based on sustainability discourse, specifically including rural and transitional rural-urban areas, with a focus on GS's recreational values. Employing a systematic review of 130 publications sourced from Web of Science, Scopus, and Google Scholar and ensuing content analysis, we identified and elaborated on 14 distinct qualities, including accessibility, connectedness, responsiveness, variety of activity, social interactions, facilities, safety and security, spaciousness, biodiversity, perceptual value, aesthetic value, and symbolic value. The interrelationships among these identified qualities are also elucidated through the content analysis.

These qualities can be utilized as a novel approach to evaluate the efficiency of PGSs in promoting sustainability.

## Highlights

• Developed a comprehensive framework for evaluating the recreational value of Public Green Spaces.
• Conducted a systematic literature review completed by content analysis.
• Introduced a novel approach to assessing the recreational value of Public Green Spaces to facilitate policy-making.
• Introduced a novel approach to assessing the recreational value for both urban and rural Public Green Spaces.

## 1 Introduction

Green spaces (GSs) play a paramount role in promoting sustainability across its all dimensions. Their contributions span all facets of sustainability, including but not limited to environmental conservation, social well-being enhancement, and economic development support. These spaces are undeniably vital in maintaining ecological balance, fostering community cohesion, and driving economic prosperity. Despite the abundance of studies exploring the significance of GSs within the sustainability discourse, a significant gap persists in the availability of comprehensive evaluation criteria. Existing studies often exhibit a predominant focus on a singular dimension of sustainability. Moreover, a considerable proportion of existing literature tends to focus predominantly on urban environments, thereby overlooking the unique sustainability challenges intrinsic to non-urban, or rural, settings. It is critical to recognize that the expectations from GSs may notably differ between urban and rural areas, particularly in the context of sustainability discourse. This gap in literature necessitates a more holistic approach to providing a GS evaluation framework that encompasses sustainability in both urban and rural GSs.

GS is comprised of vegetation and associated with natural elements and can be classified into public green spaces (PGSs) and private green spaces. Public green spaces include parks, gardens, forests, and other natural or landscaped spaces. Private green spaces are those with private ownership and private access rights, such as privately owned gardens. In this paper, our primary objective is to equip decision-makers with a robust evaluation framework aimed at facilitating the transformation of transitional regions in terms of sustainability towards a more sustainable future. To achieve this goal, our focus is specifically directed toward PGSs, which can be publicly controlled, designed, or maintained with no ownership conflict.

Research about sustainability, especially in rural areas, has predominantly focused on environmental factors, often neglecting other significant dimensions of sustainability [1]. While numerous GSs offer environmental advantages, this study emphasizes GSs with high usage potential by people. These GSs have greater sustainability potential [2], [3], [4], not only bolstering environmental health but also significantly promoting social interaction and fostering economic growth. As a result, we adopt a comprehensive approach and distance ourselves from strictly environmental viewpoints.

The recreational value of public spaces (PSs), a broader category encompassing PGSs and other non-green areas such as public buildings, streets, transportation hubs, and community centers, is crucial for urban and rural sustainability [5], [1]. This relationship can be understood through various dimensions, including environmental, social, and economic aspects. Studies have shown that recreation in PGSs is positively correlated with improved mental health, reduced stress levels, and increased physical activity [e.g., 6]. In rural areas where access to recreational facilities may be limited, PGSs become even more critical for supporting the well-being of residents [7] as a part of sustainable development [8], [9], [10]. Although rural areas are supposed to have more greenery, parks in rural areas can contribute to their sustainable development by promoting cultural values and activating the region [11].

The recreational value of PGSs is essential for promoting social cohesion and community engagement. Accessible and inclusive PGS can foster social equity by providing opportunities for all residents, regardless of age, socioeconomic status, or ability, to enjoy the benefits of nature and outdoor recreation. PGSs can serve as focal points for community gatherings and events, fostering social cohesion and a sense of place [12], [13]. In rural areas, where communities may be more dispersed, PGSs can play a vital role in bringing people together and strengthening community ties.

The recreational value of PGSs can contribute to the local economy, especially in rural areas. Tourism and outdoor recreation activities centered on PGSs can generate income for local businesses and create job opportunities. Additionally, well-maintained green spaces can enhance tourism in the urban and rural areas [14], [15], especially adding to the attractiveness of rural areas for potential residents and businesses, promoting economic development and rural vitality.

In the present study, our objective is to advance the current understanding by developing a comprehensive PGS evaluation framework with an emphasis on the recreational value. This framework considers all dimensions of sustainability and accommodates all types of PGSs in urban, rural, and transitional areas. Our methodological approach involves a rigorous systematic literature review, followed by an in-depth content analysis. The goal is to elucidate the various qualities essential for the PGS evaluation during a sustainable transition while also delineating their interrelationships. Acknowledging that progression towards a different future is inevitable, in this context, a ‘sustainable transition’ refers to the adoption of practices and modifications that foster sustainability in all its dimensions. As we navigate this inevitable shift towards a more sustainable future, our study aims to guide these transition pathways by evaluating the role of PGS in this trajectory.

In fact, our primary research aim is to answer the following central question: “Which are the fundamental PGS qualities to take into account when evaluating PGSs within the sustainability context, particularly with a focus on recreational values? To answer this, we pursued a number of sub-questions: Are there any qualities specifically applicable to either rural or urban areas? What does each main quality entail? How are these main qualities interconnected?

Decision makers and planners can leverage the outcomes of this approach to effectively monitor and manage existing PGSs, particularly in transitional areas, to foster more sustainable settlements. Additionally, they can utilize our approach in the design and planning of PGSs for future settlements. Our findings can facilitate the creation of PGSs with enhanced recreational value, thereby promoting sustainability, particularly in terms of the social and economic dimensions.

## 2 Methodology

**Figure 1:**
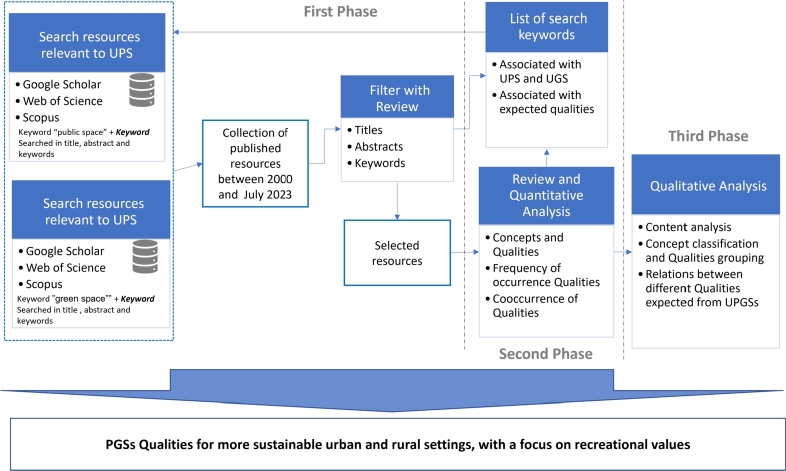
An overview of the research process.

The method adopted in this descriptive-analytical study is a systematic review of the literature followed by a qualitative approach to content analysis. Fig. 1 shows an overview of the research process.

### 2.1 Systematic literature review

For this purpose, a search protocol and review matrix were created to ensure the precision of the systematic review.

To identify relevant literature, we conducted separate searches in English for resources related to public spaces (PSs) and green spaces. We started by searching the academic databases Google Scholar, Scopus, and Web of Science using the *main terms* “public space” and “green space” combined with the keywords “sustainability” and “recreation”, in order to find sources focusing on PSs and GSs in the sustainability discourse that have a focus on recreation values. However, the majority of the resulting resources focused on urban areas. To also capture resources related to rural areas, we searched for resources with a focus on rural areas by combining the *main terms* with a list of keywords indicating rural areas. The search strings and total number of academic hits are listed in Table 1. We aimed to include all public green spaces (PGSs) in both urban and rural areas, so we excluded the word “urban” from the *main terms*. However, the majority of the identified resources primarily focused on urban spaces. To highlight this, we used the terms UPGS (Urban Public Green Space) or UPS (Urban Public Space) to indicate instances where the emphasis was solely on urban features, and PGS or GS to indicate discussions that included rural and transitional areas.

**Table 1:** Information about the initial search in Web of Science and Google Scholar and Scopus.

Search syntax	database	number of sources	time range
allintitle: green space sustainability OR recreation	Google scholar	70	2000 to Jul 2023
allintitle: public space sustainability OR recreation	Google scholar	78	2000 to Jul 2023
((TI=(“public space”)) AND (TS=(sustainability) OR TS=(recreation)))	Web of Science	52	2000 to Jul 2023
((TI=(“green space”)) AND (TS=(sustainability) OR TS=(recreation)))	Web of Science	166	2000 to Jul 2023
(TITLE (“green space”) AND (TITLE (“sustainability”) OR TITLE (“recreation”))) AND PUBYEAR > 1999 AND PUBYEAR < 2024	Scopus	66	2000 to Jul 2023
(TITLE (“public space”) AND (TITLE (“sustainability”) OR TITLE (“recreation”))) AND PUBYEAR > 1999 AND PUBYEAR < 2024 AND (LIMIT-TO (DOCTYPE, “ar”)) AND (LIMIT-TO (LANGUAGE, “English”)) AND (LIMIT-TO (OA, “all”)) AND (LIMIT-TO (SRCTYPE, “j”))	Scopus	14	2000 to Jul 2023
allintitle: “green space” Backcountry OR rural OR Village OR “Peri-urban” OR Countryside OR “Non-urban”	Google Scholar	46	2000 to Jul 2023
((TI=(“green space”)) AND (TS=(Outlying districts) OR TS=(Backcountry) OR TS=(rural) OR TS=(Village) OR TS=(Peri-urban) OR TS=(Non-urban) OR TS=(Countryside)))	Web of Science	98	2000 to Jul 2023
(TITLE (“green space”) AND (TITLE-ABS-KEY (“Outlying districts”) OR TITLE-ABS-KEY (“Backcountry”) OR TITLE-ABS-KEY (“rural”) OR TITLE-ABS-KEY (“Village”) OR TITLE-ABS-KEY (“Peri-urban”) OR TITLE-ABS-KEY (“Non-urban”) OR TITLE-ABS-KEY (“Countryside”))) AND PUBYEAR > 1999 AND PUBYEAR < 2024 AND (LIMIT-TO (SRCTYPE, “j”)) AND (LIMIT-TO (OA, “all”)) AND (LIMIT-TO (PUBSTAGE, “final”)) AND (LIMIT-TO (DOCTYPE, “ar”)) AND (LIMIT-TO (LANGUAGE, “English”))	Scopus	107	2000 to Jul 2023

The search was conducted in various disciplines, using the online databases of Google Scholar, Scopus, and Web of Science. Also, the date range of 2000 to July 2023 was applied. Then, by reviewing the titles and abstracts, a smaller subset of resources is selected. During the initial screening, we encountered some papers that fulfilled the criteria but were eventually excluded from our analysis since they were too broad and did not include any qualities for green or public spaces, or included some items that were outside the scope of our study, such as ‘produced on-site electricity’, which is not specific for assessing the qualities of public spaces, as these features may not directly address the intended objectives of our study [e.g., 16]. During the review process, we created a search keyword list to find more resources (often with the search of keyword combinations and the *main term*), which may help to clarify the concepts and their relation. In Table 2 the classification of keywords can be seen. The keywords were modified and added during the review process. After the first phase of the review and filtering the irrelevant sources, in total 98 sources are selected for the main review phase (see Fig. 6).

**Table 2:** Keywords used to identify relevant literature.

Keywords used to find relevant sources to GS and PS	green space; park; public space; rural green space; green village; urban greenery; forest; vegetation; green infrastructure; recreation area; garden; green space design; woodland; public spaces; open spaces; forest; landscape; nature; tree; natural areas; blue-green infrastructure; water
Keywords used to narrow down and limit resources related to the expectations associated with PGS	space publicness; recreation; sustainability; sustainable development; livable; happiness; activity; social interaction; accessibility, access; proximity; well-being; health; greenery density; diversity; biodiversity; safety, danger, fear; security; cultural value; water; walking; children, elderly, teenagers, vulnerable groups; aesthetic; visibility; user perceptions; spaciousness; connectivity

In the second phase, two researchers carefully assessed the sources, using a review matrix to reduce bias. Initially, all resources were reviewed and categorized as ‘included’ or ‘excluded.’ Following that, both researchers cross-compared their respective lists, debating and reconciling any differences before reaching a final consensus. This procedure entailed meticulous examination of titles, abstracts, keywords, and, in ambiguous cases, a thorough assessment of the main text. Ensuring the most comprehensive review, the final ‘included’ resources were thoroughly read by both researchers. Additional sources were also scrutinized to verify if they would introduce any new qualities to the list. The review was continued until theoretical saturation was reached, at which point the researchers concluded that no other quality expected from UPGSs of high importance in the literature existed and that additional studies would not add any new quality to the list. As a result, a total of 130 sources were reviewed. The distribution of these publications' years is presented in Fig. 7. One criterion for determining the significance of any quality is the frequency with which it is mentioned in the literature under review (see Fig. 4). The qualities were then classified and grouped as main qualities and sub-qualities using the extractable definitions and dimensions for each of the qualities from the literature. Following that, additional resources were reviewed to clarify various dimensions and details of the selected qualities resulting from the systematic review. For this review, we conducted a qualitative synthesis of key qualities associated with PGS based on the extracted data from our chosen resources. The outcomes of interest—namely, the identified qualities—were discussed and presented in a descriptive manner rather than employing quantitative effect measures like risk ratios or mean differences. As our focus was on the extraction and analysis of these identified qualities, we did not conduct a comparative analysis on the outcomes of the studies. However, we did note a degree of variability in the frequency with which certain qualities were discussed. This observational variation may reflect the prevailing focus on specific research areas at the time the studies were conducted.

### 2.2 Content analysis

As most qualities seemed to be interrelated, aiming to categorize all qualities and clarify the main PGS qualities that support sustainability with an emphasis on recreational values and to reveal the relationships between qualities, in the third phase of the research, a content analysis of a smaller subset of reviewed resources was conducted to find out important concepts and their relations. For this aim, MAXQDA [17] as software for qualitative and quantitative analysis was used. Ultimately, the finalized qualities and their interconnections are graphically represented in the form of a concept network.

To ensure accurate extraction of concepts from the selected sources, we implemented a mixture of deductive and inductive coding methods. We started with the extracted qualities from the systematic literature review as deductive codes. This involved using existing knowledge and theory to identify codes based on the research question and objectives. To clarify sub-qualities and interrelations, we used inductive coding, which involves forming codes based on the classification and generalization of text content [18], [19]. This approach allowed us to identify patterns and themes in the data and develop a more nuanced understanding of the concepts. We repeated the inductive coding process with a second researcher to ensure inter-rater reliability. Additionally, we employed two primary coding methods: “in vivo” coding, which involves selecting specific segments of the text, and “free coding,” which allows researchers to assign code names to concepts based on the context, existing literature, and findings obtained thus far in the content analysis process. Following the completion of the initial coding process, we proceeded to group these codes into broader categories, or ‘family codes’. The formation of these family codes was guided by the substantive relevance of individual codes, the frequency of recurrence, mutual exclusivity, and the thematic commonalities between them. These groups helped streamline the analysis process and provided a clearer, broader overview of the patterns and themes driving our results.

**Figure 2:**
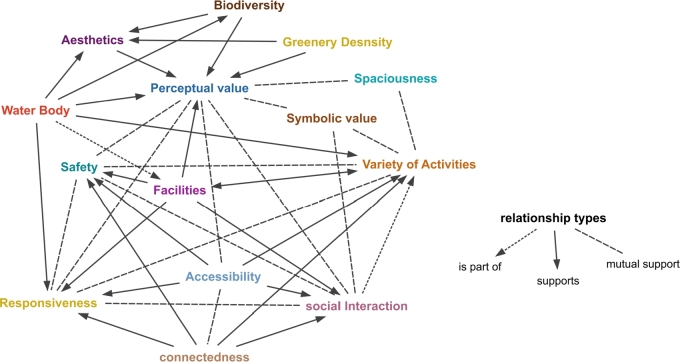
Interrelationships among PGS's Qualities Based on Literature Review and Content Analysis (We used MAXQDA Analytics Pro (version 24.0.0) (VERBI Software, 2023) for data analysis.).

These coding methods collectively contribute to the rigorous analysis and interpretation of the content. In addition, a typology of interpretation [20] is noticed to avoid biased coding and to wisely recognize the meaningful co-occurrence of codes or concepts. In other words, not all repeating nor all co-occurrence should be interpreted as a meaningful association of concepts [21]. Among the codes created, many are merged, some are modified, and many are removed in subsequent evaluations. In the end, in total 290 codes are finalized, and some main codes are classified and grouped into 21 family codes. Analytical methods, such as the co-occurring codes report, the code-primary document table, and the network drawing tool (i.e., MAXMaps) within the software, play a crucial role in defining and visualizing relationships between different qualities (refer to Fig. 2). These methods enable the identification and documentation of significant connections between different qualities of PGSs. Some of these qualities support and reinforce other qualities or have influence on some other qualities. Different types of these relationships are also illustrated in Fig. 2. Ultimately, the findings are presented and documented in the form of a comprehensive report, highlighting the important relationships uncovered during the analysis process.

## 3 Results

**Figure 3:**
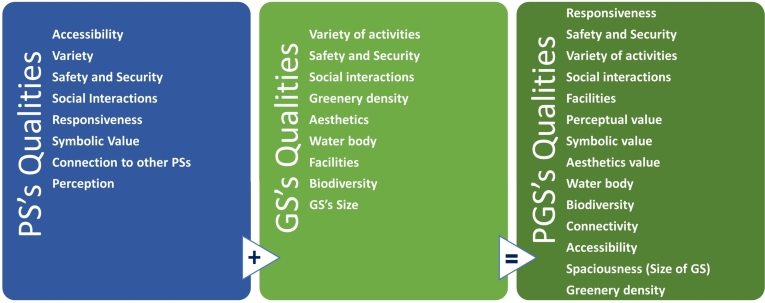
Expected qualities for public spaces and green spaces and therefore expected qualities for public green spaces.

Many resources in a variety of fields have focused on UPSs and their characteristics with many normative qualitative and quantitative criteria to evaluate them [22], [23], [24], [25]. Many tried to shed light on the relation of GSs and different dimensions of sustainability [2], [26]. Some other sources reviewed in this study, while not focusing on GSs, can help to elucidate the role of PGSs in sustainability [e.g., 13]. Recognizing PGS as a subset of PS, the majority of evaluation criteria and anticipated qualities of PS are also applicable to PGS. It's important to note, however, that PGS has its own unique typology and entails certain specific qualities. These specifics may not be relevant or hold much importance within the broader scale of PS. Consequently, in this article, we consider the anticipated qualities of both categories (see Fig. 3). This method of classification, also deployed during our literature review process, enables us to ensure precision in our review and confirm that no significant attribute is overlooked.

**Figure 4:**
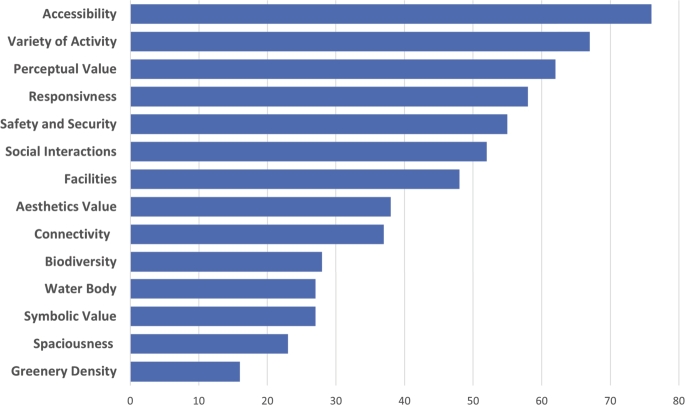
PGS's qualities and their frequencies based on systematic literature review.

### 3.1 Responsiveness

We define responsiveness as the capability to serve multiple groups of people (e.g., age groups, gender groups, and social groups). One main concern in PGS management is equity and equality in addressing different groups' needs. Spatial equity in basic service provision is a key challenge for smart development strategies and is one of the pillars of good spatial governance [27]. To enhance social sustainability, a PGS should be able to support social and cultural diversity and serve users of different ages, genders, religions, cultures, physical status, and household compositions who have varying time schedules and activity interests [28]. This social diversity indeed adds to the value of the UGS and its recreational capacity.

The main focus here is on vulnerable groups. Vulnerable groups here refer to minority populations (e.g., foreigners and ethnic minorities), the poor, the elderly, the young, and the disabled population [29], [30], [31], [32]. In rural areas, young people are particularly important as a group that migrates from the village [33], [34], while older adults are the dominant residents [35]. The loss of the youth population is a significant issue in rural sustainability [36], [37], [1] and their needs should be addressed to motivate them to stay in the rural areas. GSs can play a crucial role in responding to the recreational needs of these vulnerable populations, providing opportunities for physical activity, social interaction, community engagement, sense of place, sense of community, and promoting physical and mental health [38], [39]. Imrie and Hall (2003) observed that disabled people experience physical PSs as some obstacles. Therefore, two types of disability can be identified in PSs: medical and social. Social disability is forced on people by society and space [40]. Madanipour's research (2013) showed that social exclusion is not only for immigrants and ethnic minorities. Any characteristic that makes a person vulnerable can pave the way for social exclusion. The elderly, poor, disabled, women, children, and long-term unemployed among the native population can face the same disadvantages as immigrants and ethnic minorities [41]. This is one of the reasons why the term affordability is suggested by some scholars as a unique quality for evaluation of PGS [42]. One other source of vulnerability after race, age, and personal limitations (e.g., disability) is location, as there are some deprived or poorly served neighborhoods [32], due to the shortage of source management and allocation. Therefore, the responsiveness of PGS is an inevitable quality. PGS regulations should increasingly aim to create a space for the presence of everyone, and instead of preventing the presence of people with different social status, they should only induce the necessary codes of behavior to those present in the space [24].

### 3.2 Safety and security

We define this quality as the condition of being secure from potential harm or criminal activities. However, PGSs can also be a source of crime and therefore be unsafe, especially at night. PGSs are often seen as attractive places for doing illegal business for some criminals, and they are also seen as favorable places to sleep for the homeless. The camouflage possibilities of PGS, with their abundance of bushes and dark areas, introduce a unique factor that can contribute to their perceived higher level of unsafety compared to other public spaces (PSs) [43], [44]. Fear of crime and violence in UPSs has been one main reason for UPSs' failures in the 20th century in the shape of fear from others [45]. These unsafe PGSs can harm the neighborhood [26]. Maas et al. (2009) observed that unlike in rural areas, in urban areas PGSs can cause feelings of insecurity due to the potential hiding places they provide [44].

The lack of safety not only impacts the general public but also has a profound impact on vulnerable groups such as the elderly, women, and children [46]. The sense of safety is also important for tourists, who are vulnerable due to unfamiliar surroundings and a lack of knowledge of the local culture and language [47]. For these vulnerable groups, a sense of security in public spaces becomes even more crucial. However, it is important to note that the presence of vulnerable groups in public spaces can also serve as a positive indicator of safety, as it suggests an environment where individuals feel secure and protected [41], [48]. Safety and security of a PS is one of the most explicitly mentioned qualities by many scholars [49], [50]. And so far, many studies have been done on the fear of crime and the feeling of safety in PGS's [e.g., 51]. Many authors have cited this quality as an expected core quality of any PGS [52], [53], [54], [55], [51], [56], [43]. One central quality of PGS is that people feel safe and comfortable [57]. For different groups to visit the space and for certain activities like social interaction, intercultural interaction, recreation, and physical activity, as well as the longer stay in the space, safety, or more precisely the feeling of safety [2], [48], [58] in a PGS, is a must. These all will raise the value of that green space and its capacity to meet various demands and expectations.

### 3.3 Variety of activities

Any PS should be a neutral atmosphere that easily adapts to a variety of behaviors and provides a neutral and inductive context for self-motivated actions and a variety of purposes [59]. There are enough reasons based on reviewed resources to claim that the presence and possibility of a variety of activities is undoubtedly one quality to evaluate any PS [60], [61], [62], [63], [43], [45]. It helps to meet a variety of needs of different groups, memory making, scene of place, security, and perception of the PS [60], [45], [64]. In addition, exposure to PGSs is associated with higher physical activity [65]. The presence of a variety of activities appropriate for a variety of social groups in a PS is one solution for the inclusiveness of the space regarding the problematic dialectic of centrality and marginality, which has been the topic of many critical approaches toward PSs in recent decades [66], [67].

One of the important effects of PGSs on physical health is mainly based on the possibility of a variety of physical activity in these sorts of spaces [68], [69], [70], [71], [72], [73]. Given the importance of physical activity in the lives of people, much research has been done to explain how the design, physical configuration, and various features of PGS may influence and encourage physical activity [61], [25]. Despite the existence of studies that show more accessible PGSs are used more for physical activity, there are also some inconsistencies [e.g., 74]. Some studies show a low percentage of significant association with accessibility [75], [76]. However, it appears from studies that some other PGSs' qualities are also connected to physical activity.

### 3.4 Social interactions

According to many resources, social interaction is undoubtedly one of the main qualities that a PS should support [77], [78], [52], [79], [57], [80], [6]. PGSs provide people with opportunities to have communication as a friendly chat, especially in the neighborhood scale [12], [81]. The more a PS is allocated for neighborhood use, the more these social interactions are important in creating the social networks needed. This is also emphasized as one of the most important requirements for achieving a sustainable and resilient community [82], [83], [84], [85], [86], [87].

Leisure activities in UPSs can create social networks, and these networks are the basis for social capital formation. Civil society with the potential for citizen participation and voluntary cooperation is more likely in a community that has inherited a significant stock of social capital that can improve society's efficiency by facilitating coordinated action [88], [89]. Social capital is one of the most important aspects of sustainability in rural areas [13]. Social interaction plays a crucial role, particularly for elderly individuals, as research suggests that those with stronger social connections tend to experience lower mortality rates and enjoy better physical and mental health outcomes [90]. In PSs, there should be an opportunity for some social boundaries to be broken, for spontaneous conflicts to occur, and for individuals to mix in a new social environment [57]. The physical layout of the space, its location, and people's image of space, combined with the cultural characteristics of various ethnic groups, create opportunities for intercultural interactions and promote social cohesion [80]. In general, social interaction seems to occur more in PGSs than other PSs [91], [90], [26], [92]. Social interactions are especially significant if new links are established with people who do not belong to the close-knit circle of familiar contacts. Such contacts may enrich social capital [93], [89].

Interaction refers not only to knowing one another but also to mutual chat and brief encounters. So, interaction can be seen as a spectrum ranging from purely visual interaction as a result of co-presence in the space to friendly conversations and communal activities [57], [94]. It can start with reasons such as playing with children or events that help people to have casual conversations with strangers. The presence of certain qualities in the US, such as safety, facilities, and activities that attract people, and allow them to pause, can also draw strangers together and have a direct influence on citizen interaction and the frequency with which people get together [95]. Because the factors related to social capital are the most commonly researched drivers of rural sustainability, social interaction in PGSs is one of the most significant demands for rural sustainability [e.g., 13]. Social responsibility, participation, and sense of belonging are also mentioned as components of rural sustainability [96] that can be enhanced by social interactions.

### 3.5 Facilities

Here we use the term facility for all physical amenities and infrastructure provided within PGSs to enhance visitor experience, comfort, and usability. These facilities are designed to support various recreational, social, and practical activities in green spaces. Some common examples are toilets, benches, bins, shelters, playgrounds, or sports facilities. Considering this definition, facilities available in PGS and the quality of these facilities is another mentioned criterion by scholars [61], [57], [97], [98]. The presence of facilities in PGS appears to increase people's use (visit) of these spaces and encourage and facilitate physical activity, and social interactions. Therefore, the facilities as one of PGS's qualities, support and enhance some other qualities. In contrast, the lack of enough facilities in a PGS leads to a feeling of boredom in the space and reduces the duration and number of visits by people [45]. In addition, without well-planned facilities, the potential for a diverse range of activities within the GS may be limited. This can hinder the ability to cater to the specific needs and preferences of different groups. A well-designed and equipped GS can provide the necessary infrastructure to support a wide array of activities, ensuring inclusivity and enhancing the overall experience for various user groups.

Vierikko and others (2020) emphasized the existence of water bodies, cultural events, monuments, and memories and placed them under the category of facilities and services [78]. Some scholars focused on the management aspect of the PSs, such as cleanliness or some physical orders that have to do with facilities as well [99]. To bring people to the GS and make a pleasant perception of the space for them, facilities for their main needs, such as sitting, should be provided [100]. Some facilities are necessary for the presence of vulnerable groups. Disabled, for example, need special facilities to access and benefit from the PSs. Otherwise, they will be eliminated from the space [101], [40].

### 3.6 Perceptual value

A vital city or rural setting can have many characteristics, but the key to such a settlement is how its people perceive being invited to PSs [102]. People's perception of the PGS is key to its quality [e.g., 103]. Sense of place refers to people's perceptions and meanings based on their prior experience with a place. The place is defined as a spatial location that has been given meaning by human experiences' [104]. The PS context is not only about the physical aspect of the place but also about the people who create, occupy, and use the space. People perceive space, choose, and shape it in a mutual interaction [105]. The people's perception of the space can shape their interaction with it. Therefore, PGS should encourage people to be present in this space and be able to respond to their needs [106]. Physical order, natural composition, water body [107], safety, cleanliness, and facilities, [108] should all contribute to a pleasant perception of the space [100]. While perceptions of PSs can be influenced by personal experiences, memories, and individual learnings, there are also shared aspects of these perceptions among people. Commonalities in how individuals perceive and evaluate PSs can stem from societal norms, cultural influences, and collective experiences.

One important point about perception is that most other qualities of a PGS are highly related to people's perceptions. Perceived safety [51], [48], perceived level of biodiversity [109], [55], perceived accessibility [110], perceived publicness, perceived aesthetics [55], and perceived inclusiveness [85] are some examples.

### 3.7 Symbolic value

With symbolic value here we mean if one GS has a cultural or historical value. When a GS is or has been a place for events, has a monument or a common meaning attached, it has a high symbolic value. Whether a PS is created recently or is an old place, or even a historic place existing even before the city's or rural areas creation, matters little to most users, who are instead largely concerned with the experience it offers them [78] and consequentially with the meaning that attaches to it over time [111]. This is because PGSs provide social, cultural, and recreational services [112]. Here a place's symbolic value communicates meaning for people beyond its physical aspects. This quality questions whether there is a cultural meaning or value associated with the PGS, whether a physical cultural symbol exists in it or its immediate vicinity, and whether the PGS has a memorable meaning. In its most recent sense, it includes locations where many public memorable events occur or where many people (e.g., the elderly) simply recall certain childhood or youth memories. Some PGS users are even sensitive about changes in these spaces, such as vanishing old trees. Changes in the physical orders of a PGS can destroy people's memorable image of it [113]. Many elderly groups have a good feeling about visiting PGSs with cultural and historical value. They use these spaces to reproduce their identity and enhance the sense of place among them [45]. Local people—people except for new immigrants—follow their identity in these types of spaces and cultural events in PGS's very seriously [45]. In some cities, these events and monuments have even added to the tourism values of the UPS and are even becoming part of local government's policies [114], [22]. An old PGS that has been the place of many personal or public memories also has cultural and memorable value [105] and should not be evaluated the same as a rather newly created PGS from this aspect.

### 3.8 Aesthetic value

Aesthetic value refers to the subjective perception or appreciation of the visual, sensory, or artistic qualities of a place. It reflects the extent to which a place is considered beautiful, or visually pleasing. Green spaces are by nature a source of aesthetic perception [115]. Scenic beauty often increases recreation potential [116] and hence supports sustainability. Nature-related beauty is one of the motivations for people to visit PGSs [78]. Surveys on PGSs have shown that many people choose PGSs based on aesthetic values, which are highly associated with biodiversity, water bodies, natural landscapes, greenery density, and space design [45], [57]. Aesthetics in a PS can be in general achieved by a sense of order and pattern, rhythm, symmetry, and dominance of natural elements [117], [118]. For GSs, it is associated with harmony, multisensory, mystery and nature, and visual spaciousness and visual diversity [119]. Recreational opportunity is closely related to aesthetics and perceived aesthetics. Scenic beauty generally increases the site's recreation potential [116]. Therefore, the aesthetic quality of PGSs enhances their recreational capacity. Some studies indicate that the accessibility of a PGS only, along with the attractiveness and size of the PGS, significantly leads to more physical activity [76]. Aesthetics in a PS, however, is not as simple and as constrained as the existence of some artistic object, such as architectural marvels, but rather has to do with the tangible and immaterial circumstances that continuously and inevitably shape people's everyday lives [120], [121]. From this point of view, the more PGS is intertwined with people's everyday lives the more they perceive aesthetics in the space.

### 3.9 Water body

A water body refers to any significant natural or artificial expanse of water, such as a lake, river, sea, pond, or reservoir. The presence of water bodies in PSs has biological, economic, and social benefits and is one enhancing quality of UGSs mentioned by many scholars from a variety of disciplines, mainly under the terms urban blue spaces and urban blue-green spaces [122], [123], [100]. Urban and urban-rural areas' biological traits are enhanced by water bodies [124], and there is scientific evidence that the presence of water bodies can help preserve biodiversity [125], provide many ecological, environmental, and social opportunities for places in the vicinity [126]. People have a preference to be near water [100]. Watersheds attract people like natural magnets and can become valuable visual and recreational resources. In particular, the more visible water bodies are, the more effective they will be and the higher the enhanced value of the space [127]. Water bodies by themselves can attract the population and have recreational benefits [43], [128], and are among the qualities that can enhance the use of the PGSs and their variety of activities [61], [129]. To promote a range of physical activity, Kaczynski et al. classified water bodies as facilities and discovered that specific park amenities, such as water areas, playgrounds, and picnic places, are more crucial than other park amenities [130].

### 3.10 Biodiversity

Various fields have varying interpretations and definitions for the concept of biodiversity [131], but biodiversity here refers to the variety of life forms found within a particular GS. Biodiversity is one of the main qualities expected from PGS by many scholars from various disciplines [132], [43], [72], [77], [55], [79], [109]. Undoubtedly, biodiversity plays a crucial role in promoting environmental sustainability [133], [134]. However, its significance extends beyond just the environmental aspect, as it also contributes to various other dimensions of sustainability. The importance of the fundamental linkages between biodiversity and human health is increasingly recognized in global and regional policy development. In recent years, there has been mounting evidence linking contact with biodiversity to both physical and mental health [77], [135], [136]. Biodiversity has a high recreational impact, and a decline in biodiversity can be threatening to the quality of life of all people regardless of their social and economic status [72]. More biologically diverse PGSs are more attractive to people. There is a clear difference in motivations and enjoyment between highly bio-diverse PGSs and others [78], [137]. The importance of biodiversity in the recreational value of a place is also emphasized and measured [138]. Many recreational activities such as bird watching, hiking, camping, and fishing rely on biodiversity. The tourism industry is also influenced by biodiversity. Some studies have shown that biodiversity and structural diversity of UGS matter for perceived benefits, such as recreation and well-being [e.g., 139].

### 3.11 Connectedness

To maximize its social and health benefits, GS should not only be reachable at a walkable distance, but it should also be integrated into people's everyday journeys. People tend to shop on the way to a certain destination, meet their friends, and have the option of waiting and watching [52], [140]. Therefore, a successful GS is connected to important places in people's daily lives at a walkable distance. In addition, a minimum density with more than one main function is necessary for the vitality of a set of connected spaces [60]. Walkable spaces are those that are dense and vibrant and have a wide variety of services and activities nearby [141]. Ideally, PGSs should be situated near where people live and socialize [53]. From this standpoint, PGSs that are close to other PSs, including other PGSs, tend to achieve greater success. This condition is also referred to as “landscape connectivity” [142]. The connectedness of a PGS to other important and interesting PSs fosters social and economic sustainability. This criterion has been considered in some studies in the PGSs evaluation tools [143], [144]. An increase in social interaction and longer and more frequent visits to the PGS are among the consequences.

### 3.12 Accessibility

Accessibility seems to be the most emphasized criterion to evaluate PGS efficiency in both urban and rural areas [e.g., 145]. The majority of scholars in all reviewed references in this research have mentioned the importance of accessibility, especially to PGSs, to foster sustainability [145], [146], [147]. It is also the most consensual condition among scholars for considering a US public and has an undeniable role in promoting other expected qualities of space [148]. Although numerous studies have measured levels of access to different PGSs [42], [149], there seems to be no consensus on how to measure PGS access [145], [150]. Some have emphasized mobility as well [e.g., 151]. However, accessibility refers to the distance from a resident's home to a PGS in addition to how safe and easy the person can get to it [152]. Access to public transportation is, therefore, an important factor in determining the accessibility of PGS for different social groups [97], [42]. Although accessibility is undoubtedly the core quality of a PGS, some studies show that access to PGS alone is not enough to increase citizen use [153]. Also, different types of distances are considered in research experiences; some papers emphasized route network distances and access points [e.g., 154], as it is more realistic and precise. And some others are content with Euclidean distance [e.g., 155]. Regardless of which approach is taken, a variety of distances have been chosen [130], [153].

When it comes to PSs accessibility, not only physical access but also social access (i.e., accessible for all social groups), access to activities, access to information [41], [60], and visual accessibility (i.e., being visible from the outside of the space and different parts of the space) should be considered. The visibility of various activities, facilities, and different parts of PGS not only supports these activities and invites people to use the space, but also supports the security of the place at different times of the day [60]. The visual permeability of a PS can enrich the public domain and inform people about the possibilities and facilities inside the space. This is usually associated with physical accessibility [156].

### 3.13 Spaciousness

The visitor's perception of the size and shape of the GS, as well as the associated comfort, is referred to as spaciousness. Scholars primarily believe that PGS should be large enough to provide users with a variety of options while remaining appealing to them [157], [136]. It seems that people are more satisfied in larger PGSs and find them more appropriate for a variety of activities [61], [76], [158]. However, there is a distinction to be made between the size of a GS and its spaciousness. Although many studies looked at PGS size as a factor in improving people's use [e.g., 159], many others noticed to point out that not all large PGSs are more attractive [e.g., 98]. This is especially more important in great cities and megalopolises [e.g., 42]. Some findings indicate that there may be an optimum range for GS's size. PGSs that are smaller than a specific size or larger than a size are not associated with citizen satisfaction [61], [74]. Furthermore, the larger the PGS, the more space management will be crucial.

### 3.14 Greenery density

In addition to ecological benefits, the existence of vegetation and density of the vegetation in a PS increase people's rating of that space [160], [159]. Many scholars have mentioned the greenery density as an important quality to value PGSs, and some of them also have mentioned its positive effect on the perceived aesthetic [155], [49], [79], [161]. [159] approve the positive role of greenery density in people's perception but mention that there is no proof of a positive effect on the activity in the space based on studies so far. Some scholars also believe that if the greenery density is high, it can lead to a sense of fear and insecurity due to the camouflage possibility and less light in some parts of the GS at night [43], [162]. However, when vegetation is well integrated into landscape design, PGS can have higher vegetation density without necessarily appearing to be unsafe for people [162]. Also, depending on the vegetation types, different activities can be exerted [163]. Therefore, the greenery density is considered one important quality to evaluate PGSs. However, it should be well managed and designed. Although there is a belief that PS evaluations can be done regardless of PS governance, including space management and design governance [e.g., 60], considering the process of production and maintenance of PGSs can lead to more efficient PGSs.

### 3.15 Conceptual relation between PGS qualities

It is inferred from the literature that main PGS qualities are interrelated and exhibit connections among themselves. Notably, some of these qualities mutually support and reinforce one another. To investigate these interrelations, a more in-depth investigation was performed in this research by content analysis of texts. A good state in the quality of facilities, for example, can support activities and social interactions. Some qualities can be considered as part of other qualities. Visibility, for instance, can be considered a part of the main quality of accessibility and thus is not mentioned as an independent quality. Social interaction, on the other hand can be considered as one activity but due to its importance, is considered as one distinct quality. Some qualities are mutually interrelated and have mutual effects (e.g., safety and variety of activities seem to be positively associated). The following part of this section includes some examples of these analyzed texts.

Scholars unanimously agree that accessibility is the essential quality of any PGS, leaving no room for dissenting claims. In addition, many have mentioned the association between accessibility and other important qualities. E.g., “Proximity to public open space, such as parks and other green spaces, …, and people are more likely to use such space for physical activity [164].” or “The availability [accessibility] of green spaces … enhances the feeling of social safety …, increases social interaction … [115]”. In some other resources the relation between age, social groups, activities, and accessibility is noticed; e.g., “When forests are recreational green spaces, the WTP [willingness to pay] is positive for more affluent socio-professional category (SPCs) and higher for the oldest households but negative for less affluent social groups, Forest amenities, such as hiking and biking paths, positively affect the WTP for almost all SPCs, especially the youngest household [165].” Special activities make the green space more responsive to some social groups (e.g., age, gender, economics, etc.). Hence, accessibility encompasses not only quantitative aspects but also qualitative dimensions, which vary across different UPGS and social groups.

The relation between perception and other qualities is another frequently mentioned issue in sources. E.g., “The scope of “safety and security” includes perceived safety and security [149].” “The level of use is not just a function of perceived safety but also reflects attitudes regarding recreational and aesthetic value. Perceptions of the benefits of trees and landscape can vary considerably among different ethnic groups [166].” or “...measures of perceived proximity were more likely to be significantly associated with physical activity than measures of objective proximity.” [26] Regarding the relation between social interaction, accessibility, and connectedness one resource mentions: “... the connectedness of space, ease of accessibility, distance, quality, attractiveness, and maintenance, are features of the physical environment contributing to increased social interaction” [2]. The triangle of safety, perception, and social interaction is another frequently mentioned relation of these qualities. E.g., “Negative interactions are often related to discrimination and make users uncomfortable or lead to anger and physical violence … Fear is created not only by perceived discrimination; many studies show that public spaces themselves are perceived as spaces of fear … [57].” The effect of activities and facilities on the quality of social interaction can also be perceived in many texts. E.g., “Interactions in public spaces can be initiated by a third party or an object, … [this] helps people to strike up casual conversations with unknown people. The presence of an event or amenity, or facilities that attract a lot of people, can also draw strangers together [95].”

The aesthetics and biodiversity quality of PGS are highly interrelated with individuals' perceptions. The perceived aesthetics and biodiversity are what should be considered in urban greenery planning to have more adaptive results with people's needs. E.g., Some scholars believe that “…there is an urgent need for greater understanding of the complex relationships between human aesthetic experience, well-being, and actual or perceived biodiversity [55].” “…in general, water elements which have different characters are used for two main purposes; “aesthetic” and “functional”.

Furthermore, there exists a significant correlation between the presence of a water body and aesthetics, as well as between the water body and UPGS activities. Several findings suggest that the inclusion of a water body, along with specific amenities, can positively influence the occurrence and engagement in activities. E.g., Schipperijn and others have written: “…We furthermore found positive associations for the presence of a water feature (i.e., lake, stream), a pleasant view to the outside of the UGS, a bike rack, or a parking lot [with physical activity] [61]. “The relation between size and other UPGS qualities and the importance of the concept of spaciousness can be perceived from some texts. E.g., “…scenic beauty generally increases a site's recreation potential. Although area size is important, informal UGS such as allotments and neighborhood green are found to be equally as attractive for recreational purposes as formal UGS such as parks [167].” There are many examples of texts that indicate the relationship between security and safety in social interactions. E.g., “This is important, as the reasons why people are afraid of green space might not have environmental origins at all. Several studies in green spaces have mentioned a lack of social relations or absence of park personnel … [162].”

There also seems to be a relationship between social interactions and well-being. e.g.,”... our discussion primarily focuses on social interactions among residents that influence health outcomes.... Many studies demonstrate how social cohesion can influence a range of factors that are linked to physical and psychological well-being.” [26]

## 4 Discussion

We started our study with one main question: “Which are the fundamental PGS qualities to take into account when evaluating PGSs within the sustainability context, particularly with a focus on recreational values?”. In this study, we could find out 14 qualities of accessibility, connectedness, responsiveness, variety of activity, social interactions, facilities, safety and security, spaciousness, biodiversity, perceptual value, aesthetic value, and symbolic value to evaluate PGSs from the perspective of recreational value and in the sustainability context. We also could explain each quality and shed light on the relationship between them. We limited our systematic review to literature published between 2000 and July 2023 in order to include more recent and thorough information. However, we later removed this restriction in order to provide a more detailed explanation of each quality and its relationship. We also limited our search to the sources in English, the most common scientific language for academic studies. Through a content analysis of a limited selection of our reviewed sources, we tried to explain different aspects of each quality and the relationship between these qualities. Including more sources in the content analysis process may result in a more thorough grasp of these qualities and relationships. We believe explaining each quality in more details, like releasing other constraints in our study, would exceed the scope of one paper.

To our knowledge, this is the first study to provide a framework that includes key qualities for evaluating PGSs in both rural and urban settings from a recreational and sustainability perspective. While existing frameworks focus on urban-specific qualities, we intentionally strived for a balanced view in the literature review process, in spite of the challenge due to an urban-centric bias in available literature. While existing frameworks do not provide a clear overview of PGS's qualities interrelationships, our combined approach of a systematic literature review and content analysis successfully enabled a detailed exposition of each quality along with their interrelationships—a first for this sphere of research. Researchers, decision-makers, and planners can use our comprehensive framework widely to assess PGSs. To the best of our knowledge, while recreational values of PGSs have been explored in previous research, our study brings a novel contribution by integrating these aspects into a comprehensive framework of evaluating PGSs for more sustainability for the first time. Compared to existing criteria in other studies, our tool is more precise and thorough. It encompasses all major qualities and their various facets, thus providing a more definitive understanding of how each quality bears relevance to others.

Our proposed PGS evaluation framework has broad potential applications. It could play an important role in future decision-making processes in both rural and urban contexts by offering an all-inclusive basis for evaluating and comparing PGSs in order to guide resource allocation and improve the future of PGS in accordance with sustainability criteria. Recognizing its potential to expand our understanding of PGSs in light of sustainability criteria, we believe this framework could heavily influence future studies in the same field. Future studies could build upon our work by examining the framework in distinct geographical contexts or using sources in other languages to create a precise, locally applicable evaluation framework. We also recommend that future studies consider incorporating a wider selection of sources in the content analysis to gain more in-depth insight into each quality and the interconnectedness between the principal attributes of PGSs. The potential of this research to guide future work is vast, and we look forward to the progress it can contribute to in the ongoing discourse on sustainability.

## 5 Conclusion

In this article, we discussed that there are various expectations of PGSs in different disciplines regarding leveraging sustainability, and there is a necessity for clarifying the PGSs qualities expected in each discipline to have a practical, comprehensive method in hand for their evaluation. We focused on those GSs that can be used by people and therefore can support all dimensions of sustainability. We also emphasized the recreational value of these spaces.

In order to answer our research questions, we conducted a systematic literature review and a content analysis. As a result, we extracted 14 qualities that PGSs should enhance to support recreational value and, hence, sustainability. Further review of resources yielded two outcomes. First, it became clear that the differences in urban and rural areas lie not in the main extracted PGS qualities but in their details. Second, many aspects of these qualities are made clearer, opening the door to a more realistic measurement of these qualities. Additionally, we were able to depict the relationships between these qualities.

Given the pivotal role of PGSs in advancing numerous sustainability objectives, coupled with the shortcomings of current measurement methodologies that often solely target urban areas or single sustainability dimensions, our framework holds substantial promise.

A remarkable attribute of our methodology is the emphasis put on the recreational value of PGSs. While there are existing indices meant for evaluating the success of PSs, there has been a distinct need to determine specific criteria for evaluating the recreational worth of PGSs. Our findings offer instrumental guidance to decision-makers and planners working to improve PGSs, particularly from a recreational standpoint. This increased focus on recreation not only elevates public engagement, well-being, and satisfaction but also fosters enhanced sustainable development within social and economic domains. Using our framework as a diagnostic tool, practitioners can effectively assess the current condition of existing PGSs, identify areas of potential enhancement, and implement improvements that are aligned with sustainability principles. This capability underpins PGSs' active preservation and long-term progression. Beyond evaluating existing PGSs, our framework serves as a strategic guide in planning future PGSs. It provides essential support in resource allocation, ensuring they are utilized optimally to establish high-value, successful PGSs. Ultimately, this promotes the development of sustainable urban and rural settlements, benefiting communities both now and into the future.

**Figure 5:**
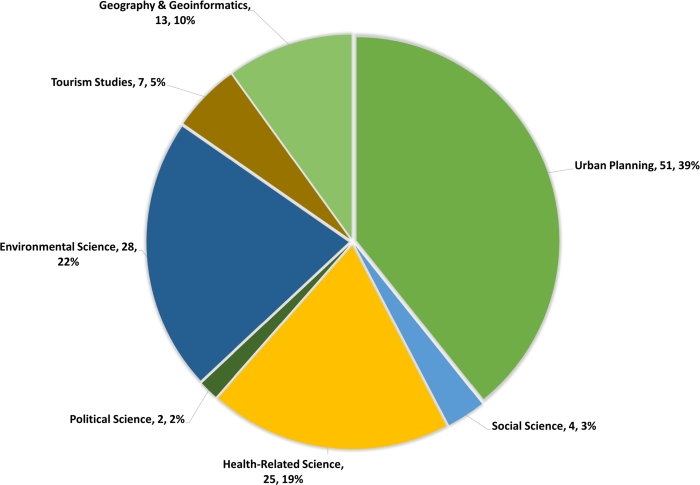
Number and percentage of reviewed references from each discipline.

This article reviews 130 resources in English from various disciplines. The distribution of these references across each discipline is illustrated in Fig. 5. This study focuses on the recreational needs of people. Reviewing more papers from other specialized fields or in other languages may yield different results. A literature review with a different focus may also reveal different qualities expected of UPGSs. In our systematic review, we noted some diversity in how frequently qualities were addressed across studies. This could stem from research focus variations or geographic and cultural differences. Future work may explore these variations further, but this was beyond our current review's scope.

**Figure 6:**
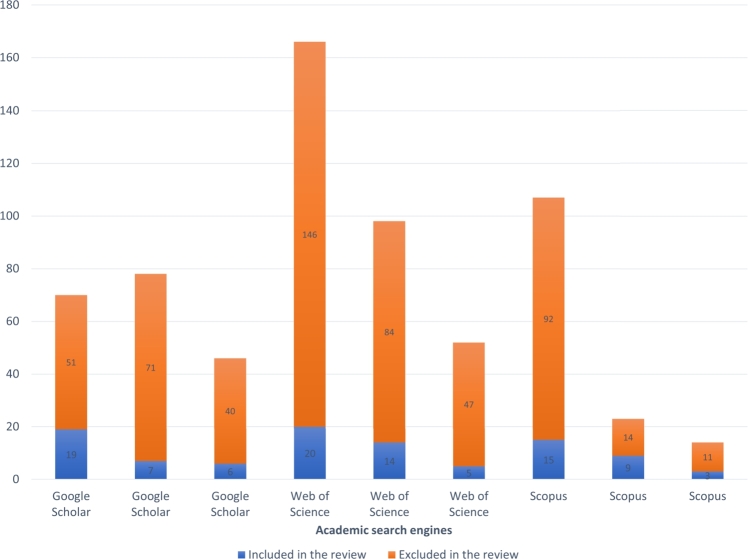
Number of included and excluded sources from each search engine during the review process.

**Figure 7:**
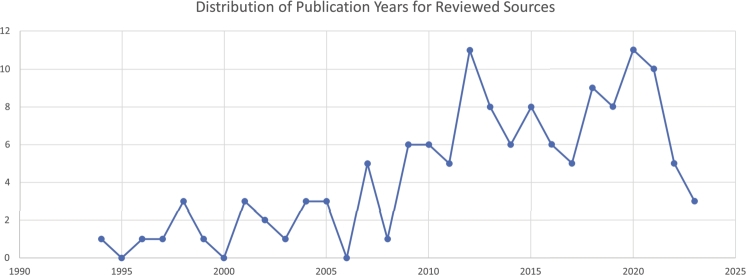
Distribution of publication years for reviewed sources.

## CRediT authorship contribution statement

**Amirmohammad Ghavimi:** Writing – review & editing, Writing – original draft, Visualization, Software, Resources, Methodology, Investigation, Formal analysis, Data curation, Conceptualization. **Frank Schuessler:** Writing – review & editing, Validation. **Roland Pesch:** Writing – review & editing, Validation, Supervision, Software.

## Declaration of Competing Interest

The authors declare the following financial interests/personal relationships which may be considered as potential competing interests: Roland Pesch reports financial support was provided by Lower Saxony State Ministry of Science and Culture with the grant number 11-76251-1840/2021. If there are other authors, they declare that they have no known competing financial interests or personal relationships that could have appeared to influence the work reported in this paper.

## Data Availability

All the data supporting the findings of this systematic review and content analysis are derived from accessible articles indexed in scientific databases, dissertations, and books. The specific search engines, search terms, and number of retrieved papers are detailed in the methodology section of this paper. The extraction and analysis of the concepts (qualities) were conducted using MAXQDA. A selection of these sources, including used sources for a deeper understanding of concepts and their relationships, have been acknowledged and listed in the references section of this article. All data utilized is derived and synthesized from existing literature, and no new, previously unpublished primary data was produced in the research process.
